# Automated Manufacturing Processes and Platforms for Large-scale Production of Clinical-grade Mesenchymal Stem/ Stromal Cells

**DOI:** 10.1007/s12015-024-10812-5

**Published:** 2024-11-15

**Authors:** Magdalena Strecanska, Tatiana Sekelova, Veronika Smolinska, Marcela Kuniakova, Andreas Nicodemou

**Affiliations:** 1 Institute of Medical Biology, Genetics and Clinical Genetics, Faculty of Medicine, Comenius University, Sasinkova 4, Bratislava, Bratislava, 811 08 Slovakia; 2 National Institute of Rheumatic Diseases, Nabrezie I. Krasku 4, Piestany, 921 12 Slovakia; 3 GAMMA-ZA, Kollarova 8, Trencin, 911 01 Slovakia

**Keywords:** Mesenchymal stem/Stromal Cells (MSCs), Cell Therapy, Good Manufacturing Practice (GMP), Large-scale Production

## Abstract

Mesenchymal stem/stromal cells (MSCs) hold immense potential for regenerative medicine due to their remarkable regenerative and immunomodulatory properties. However, their therapeutic application requires large-scale production under stringent regulatory standards and Good Manufacturing Practice (GMP) guidelines, presenting significant challenges. This review comprehensively evaluates automated manufacturing processes and platforms for the scalable production of clinical-grade MSCs. Various large-scale culture vessels, including multilayer flasks and bioreactors, are analyzed for their efficacy in MSCs expansion. Furthermore, automated MSCs production platforms, such as Quantum^®^ Cell Expansion System, CliniMACS Prodigy^®^, NANT001/ XL, CellQualia™, Cocoon^®^ Platform, and Xuri™ Cell Expansion System W25 are reviewed and compared as well. We also underscore the importance of optimizing culture media specifically emphasizing the shift from fetal bovine serum to humanized or serum-free alternatives to meet GMP standards. Moreover, advances in alternative cryopreservation methods and controlled-rate freezing systems, that offer promising improvements in MSCs preservation, are discussed as well. In conclusion, advancing automated manufacturing processes and platforms is essential for realizing the full potential of MSCs-based regenerative medicine and accomplishing the increasing demand for cell-based therapies. Collaborative initiatives involving industry, academia, and regulatory bodies are emphasized to accelerate the translation of MSCs-based therapies into clinical practice.

**Graphical Abstract:**

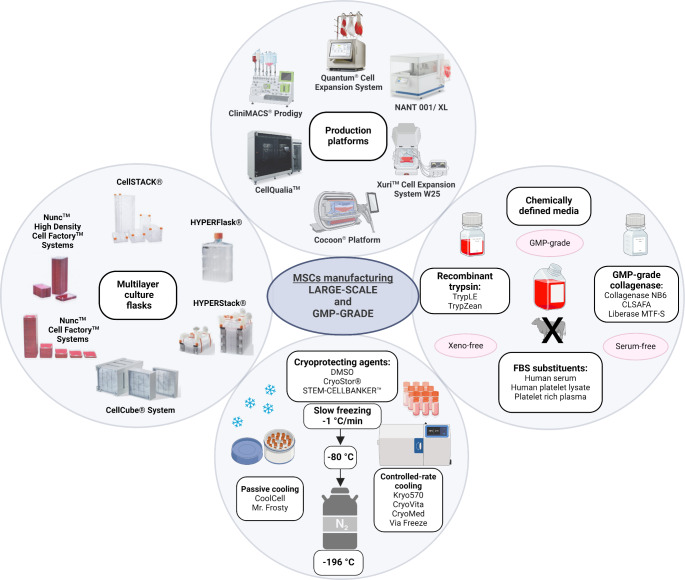

## Introduction

Mesenchymal stem/stromal cells (MSCs) are a type of multipotent adult stem cell found in various tissues, including bone marrow, adipose tissue, and umbilical cord. They possess remarkable self-renewal and differentiation capabilities, enabling them to generate various cell types such as osteoblasts, chondrocytes, or adipocytes. Moreover, MSCs secrete a variety of bioactive molecules, including growth factors, cytokines, and extracellular vesicles, which contribute to tissue repair and regeneration. These paracrine factors exert anti-inflammatory, anti-apoptotic, and pro-angiogenic effects, promoting tissue healing in various injuries and diseases. In addition, one of the key features that make MSCs attractive for therapeutic applications is their immunomodulatory properties. MSCs have been shown to suppress immune responses by inhibiting the proliferation and function of various immune cells, including T cells, B cells, macrophages, and dendritic cells. This immunomodulatory effect makes MSCs promising candidates for the treatment of various autoimmune diseases, graft-versus-host disease (GVHD), and inflammatory syndromes. As a result, MSCs offer a promising avenue for regenerative medicine, tissue engineering, and treatment of autoimmune diseases, consequently holding great potential for addressing unmet medical needs [1, 2].

Because the production of MSCs is considered ATMPs (Advanced Therapy Medicinal Products), their application is controlled by a specific regulatory framework. The MSCs production requires large-scale cell expansion systems in compliance with current Good Manufacturing Practice (GMP) standards. GMP guidelines are stringent regulations established by government regulatory authorities, such as the Food and Drug Administration (FDA) in the United States or the European Medicines Agency (EMA) in Europe, to ensure the quality, safety, and efficacy of medicinal products, including MSCs based products [3]. Taking into consideration the fact that MSCs-based therapy involves the administration of living cells that are not capable of filtration or sterilization afterward, the first GMP prerequisite for MSCs manufacturing is an environment with controlled sterility and multi-level aseptic protection solutions. Together the clean room facility, the sterile gowning, laminar flow hoods, and closed cultivation systems are involved to restrain the number of particles in the air and to avoid contamination of the final product. Last but not least, a well-trained and organized staff that understands the manufacturing process is also important to not harm the patient [4].

The production process of MSCs-based therapy involves steps such as donor and MSCs source selection, cell isolation and expansion, quality control, and further transplantation of expanded cells. One of the key challenges in utilizing MSCs for therapeutic purposes is the ability to expand them in large quantities while maintaining their properties and functional characteristics. Generally, the initial frequency of MSCs in native tissues and collected biological material is very low – it was reported less than or about one MSCs per 10^4^–10^5^ mononuclear cells in bone marrow, or per 10^2^–10^3^ cells from lipoaspirate respectively [5]. Traditional in vitro expansion of MSCs to a clinically relevant yield (millions to hundreds of millions of cells) is often extremely time-consuming, labor-intensive, and requires a lot of incubator space [6, 7]. Therefore, it is not only inefficient but may also compromise the quality of expanded MSCs. Importantly, previous studies reported the decrement proliferation and differentiation capacity of MSCs in late passages [8].

Another integral part of the MSCs-manufacturing process is the quality control of the final product. This includes assays for cell viability, phenotype markers, differentiation potential, and absence of microbial contamination to assess the purity, identity, potency, and safety of manufactured cell products. According to the International Society for Cellular Therapy (ISCT) MSCs must meet the following aspects: (I) plastic adherence, (II) positivity for the expression of cluster of differentiation (CD) molecules like CD105, CD73, and CD90, and lack expression of CD45, CD34, CD14, CD11b, CD79α, and Human Leukocyte Antigen (HLA)-DR, (III) differentiate to adipocytes, chondrocytes, and osteoblasts in vitro [9]. Nevertheless, to ensure the MSCs´ safety and functionality, additional testing of immunomodulatory activity and genome stability should be performed [10, 11].

## MSCs Production Platforms

**Fig. 1:**
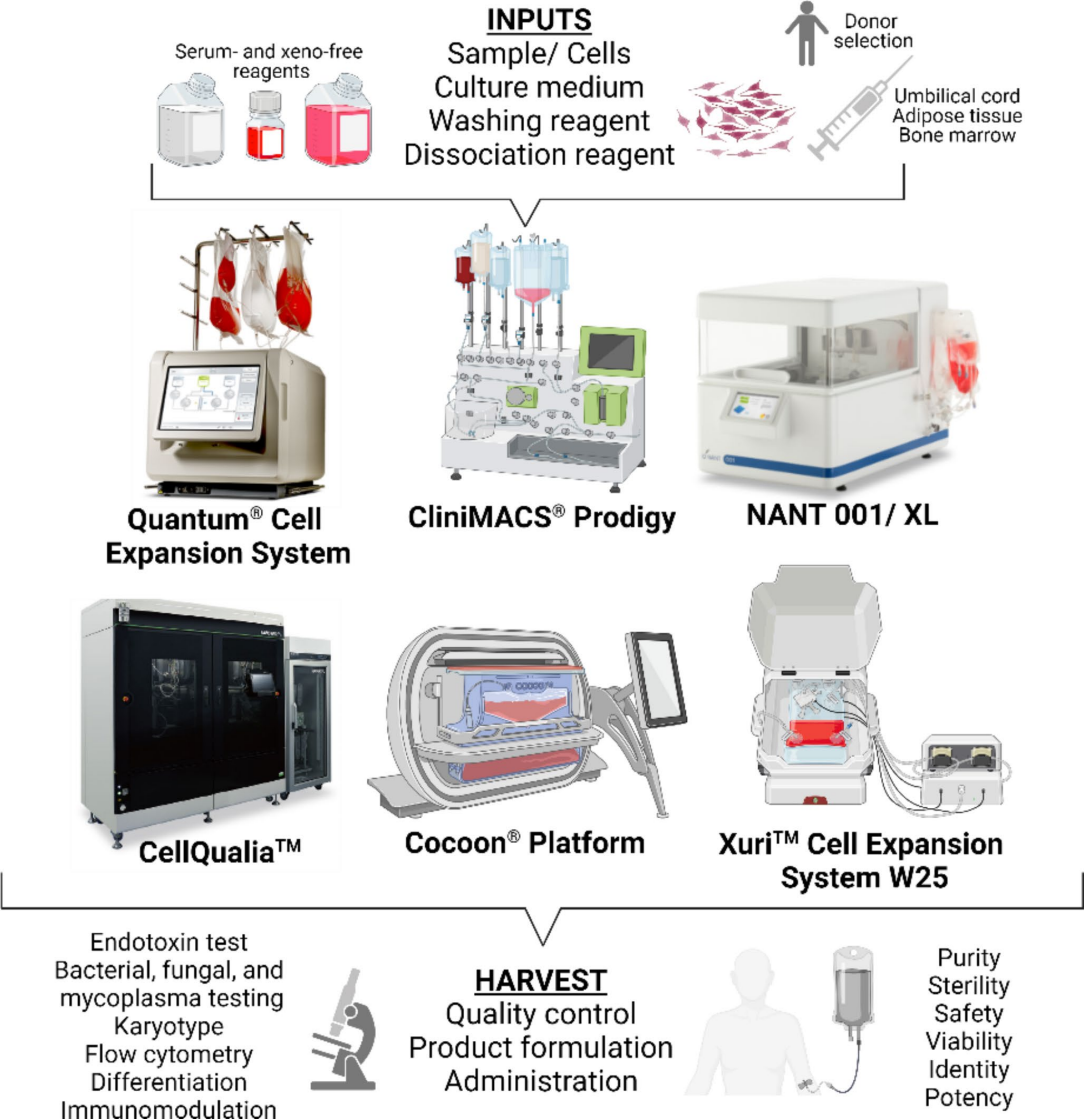
Complex overview of bioprocessing strategies for large-scale production of clinical grade mesenchymal stem/ stromal cells (MSCs). MSCs expansion platforms (Quantum ® Cell Expansion System, CliniMACS Prodigy ® , NANT001/ XL, CellQualia ™ , Cocoon ® Platform, and Xuri ™ Cell Expansion System W25) require the following inputs: sample or isolated cells, culture media, washing reagent (phosphate buffer saline), dissociation reagent (trypsine, TrypLE), and optional supplements. Downstream processes such as cell harvest, quality control (purity, identity, potency, and safety of final product), cryopreservation, and product formulation are also essential parts of clinical-grade MSCs manufacturing. Created with BioRender.com

The bioprocessing strategies for large-scale production of clinical-grade MSCs is a complex procedure with a high requirement for a closed and sterile environment. Consequently, MSCs processing must take place in a dedicated and GMP-compliant facility that minimizes the risk of contamination by physical barriers and air filtration. In response to the customer needs within this field, several manufacturers are developing and introducing new technologies for automatic and close cell cultivation, such as the Quantum^®^ Cell Expansion System (Terumo BCT), CliniMACS Prodigy^®^ (Miltenyi Biotec), NANT001/ NANT XL System (VivaBioCell), CellQualia™ (Sinfonia technology), Cocoon^®^ Platform (Lonza), or Xuri™ Cell Expansion System W25 (Cytiva) (Fig. 1). The utilization of bioreactors holds immense potential in expanding access to cell-based therapies, especially for centers lacking on-site GMP-compliant laboratories. These bioreactors provide a controlled environment for cell culture and expansion, ensuring consistency, reproducibility, and quality of cell manufacturing processes. By employing bioreactors, centers without GMP facilities can still produce clinical-grade cells, thereby broadening the availability of cell-based treatments to more patients [12].

The Quantum^®^ Cell Expansion System developed by Terumo-BCT represents an alternative strategy of automatic and closed cell cultivation utilizing a hollow fiber bioreactor. Additionally, this device offers continuous exchange of culture medium at a rate that suits the cell culture process. The bioreactor itself provides an area of 21,000 cm^2^ which is equivalent to 120 T-175 flasks. Of note, the hollow fibers of this bioreactor must be coated with an adhesive substrate, like fibronectin, vimentin, or cryoprecipitate (typically derived from the blood of multiple donors) before the cell seeding. To date, up to 25 experimental studies have been testing the Quantum^®^ Cell Expansion system for adult human MSCs expansion, making it the most widely used bioreactor system for adherent cells [13]. Moreover, the Quantum^®^ was shown to be superior to manual flask-based culture when comparing the yield and population doubling of MSCs derived from adipose tissue (AT-MSCs) and thus is suitable for large-scale production. Additionally, the substitution of fetal bovine serum (FBS) with human platelet lysate (hPL) as a growth supplement significantly enhances the expansion of AT-MSCs within the Quantum^®^ while sustaining their quality [14]. For instance, a 7-day expansion of thawed MSCs derived from bone marrow (BM-MSCs) (at passage (P) 2) loaded to Quantum^®^ at a density of 20 × 10^6^ resulted in a yield of 100–276 × 10^6^ [15]. Furthermore, several studies have demonstrated the preservation of immunomodulating function of Quantum^®^-expanded BM-MSCs that are capable of suppressing the activation of T-lymphocytes in vitro [16, 17]. In addition, the Quantum^®^ system can be directly connected to any gases and their combination, thus providing either normoxic or hypoxic microenvironment. Hanley et al. observed lower productivity of flask and normoxia-cultured BM-MSCs compared to their Quantum^®^ and hypoxia-expanded counterparts. On the other hand, it’s probable that the enhanced monitoring of lactate and glucose levels, coupled with the constant supply of fresh media within the Quantum^®^ bioreactor, fostered superior survival and overall growth. Generally, the Quantum^®^ reduced the number of needed passages (to half) and open manipulation (from 54,400 to 133 steps) compared to flask-based propagation. Moreover, their study also claims the therapeutical efficacy of Quantum^®^-expanded BM-MSCs in rat models of ischemic stroke [18]. Another study revealed the healing capacity of Quantum^®^-manufactured MSCs derived from umbilical cord tissue (UC-MSCs) and BM-MSCs transplanted to mice with induced joint surface defect of the knee [19]. Clinical trials employing the Quantum^®^ bioreactor system explore treatments for conditions like GVHD, type 2 diabetes, Parkinson’s disease, high-grade gliomas, ischemic heart disease, stroke, and acute respiratory distress syndrome. Utilizing various MSCs types, including BM-MSCs, AT-MSCs, or neural MSCs these trials demonstrate the safety, tolerability, and effectiveness of cells produced with the Quantum^®^ system across different dosages and administration routes [13].

CliniMACS Prodigy^®^ device from Miltenyi Biotec utilizing the ACC (Adherent Cell Culture) process and appropriate tubing set (TS730), enables automatic MSCs isolation (by density gradient centrifugation of bone marrow sample), inoculation, cultivation, media change, propagation, and harvesting. Godthardt et al. showed the large scale and clinical grade efficiency of this system together with the use of specific MSC-Brew GMP medium. Additionally, they claimed that primary tissue-isolated BM-MSCs as well as single-cell suspensions of AT-MSCs or UC-MSCs can be processed by CliniMACS Prodigy^®^ [20]. Additionally, it was reported that a 10-day long procedure using 1-layer CellSTACK^®^ was able to generate more than 100 colonies comprised of 29 to 50 million MSCs (passage zero – P0) isolated from horse, electro-acupuncture-mobilized samples of peripheral blood. Isolated MSCs possess characteristic fibroblast-like morphology and MSCs phenotypic features. Importantly, the Prodigy system yielded significantly higher numbers of MSCs harvested at P0 compared to manual protocols with purity improving through subsequent manual passages, reaching 95% positivity for CD73, CD90, and CD105 in P3 [21]. In the manufacturer study, human bone marrow-mononuclear cells were initially seeded in a 1-layer CellSTACK^®^ Chamber. These cells were then washed, cultured, and expanded into three 5-layer CellSTACK^®^ Chambers using MSC-Brew GMP Medium within the CliniMACS Prodigy^®^ System. For comparison, a manual expansion process was performed using T175 flasks. After 14 days of expansion, the CliniMACS Prodigy^®^ System yielded 3.8 × 10⁸ BM-MSCs (passage 2), while the manual process yielded 4.5 × 10⁸ cells at the same passage, indicating similar cell numbers between the automated and manual methods [22]. However, the platform complexity and high consumable prices make the CliniMACS Prodigy^®^ a costly manufacturing option, especially for small-scale MSCs propagation.

NANT 001 or XL systems provide another platform that contains either one CellSTACK^®^ (636 cm^2^) or two interconnected (T-175 and 5-layer flask of 3180 cm^2^) cell culture containers in the case of the XL system. Unlike the CliniMACS Prodigy^®^, this system does not require an external incubator. On the other hand, it has a lower capacity, however sufficient for autologous production of small to medium batches. Moreover, it allows smart and remote monitoring of in-process control information (e.g., temperature, pH, confluence, and detachment of cells). The NANT Cartridge is a closed system, composed of four easily connected sections via sterile connectors [23]. Fitzgerald et al. tested the efficiency of the NANT 001 system for GMP-compliant production of autologous AT-MSCs from healthy donors. The manual and automated processes were performed in parallel and cells from processed stromal vascular fraction (SVF) were placed into one 636 cm^2^ CellSTACK^®^ culture chamber at a density of 4,000 cells/cm^2^. The culture medium with a total volume of 150 mL consisted of an alpha modification of Minimum Essential Medium (α-MEM) supplemented with 5% hPL, and 1U/mL heparin. The cultured samples were washed with 150 mL of phosphate-buffered saline followed by the media exchange at days 1, 3, and at 50% confluence. Expanded cells were harvested by 50 mL of recombinant TrypLE solution 24 h after reaching 90% confluence. Data from three validation runs revealed comparable cell yield (average 2.7 × 10^7^) and viability (> 90%) of manual and automated processes lasting for 6–8 and 7–9 days, respectively. Moreover, both expanded AT-MSCs possess MSCs phenotypic characteristics and similar differentiation capacity to adipocytes and osteocytes [12].

CellQualia™ represents another intelligent cell processing system engineered by Sinfonia technology. This system enables close and automated manufacturing of MSCs using two cell culturing units and auto-passaging from 1- to 5- (CellSTACK^®^) and 36-layer (HYPERStack^®^) chamber. The build-in process of analytical technologies ensures a high level of quality control by in-line (temperature, CO2, morphology, pH, glucose, and lactate) and off-line analysis of automatically collected samples. By monitoring lactate levels over time, it is possible to indirectly assess cell growth and proliferation kinetics. Once lactate levels reach a certain threshold indicating the optimal cell density, the system indicates the need for auto-passaging. Moreover, the accumulation of kynurenine and the maintenance of low levels of 2-aminoadipic acid indicate cell retention in undifferentiated status throughout the procedure. Hosoya et al. confirmed the plastic adherence and phenotypic characteristic of CellQualia™ serially propagated MSCs that were positive for MSCs specific markers: CD105, CD73, and CD90. However, the study lacks the evaluation of MSCs negative markers and their differentiation capacity [24]. Starting from 2 × 10^6^ the company indicates MSCs yield corresponding up to 1 × 10^8^ cells after 2 passages and 6 days in culture [25].

The Cocoon^®^ Platform introduced by Lonza was designed to address the manufacturing requirements of autologous (patient-scale) cell therapies. This system uses a closed single-use disposable cassette offering either planar surface or volumetric space for culturing anchor-dependent or independent cells. Within the cassette, a thermal barrier segregates warm and cold regions, ensuring optimal conditions for cell culture (37 °C) and reagent storage (4 °C). Additionally, the cassette is equipped with pH and dissolved oxygen sensors, enabling real-time data recording during the manufacturing process. This platform is customizable and easy to use and requires minimal operator interaction, however, its wide implementation at this time is unclear [26, 27].

The Xuri™ Cell Expansion System W25, developed by Cytiva, represents a leap forward in bioprocessing for cell therapy. The system accommodates unidirectional WAVE™ rocking technology and various culture volumes (from 300 mL to 25 L), suitable for either small-scale research or large-scale clinical production. The gentle rocking motion provided by the WAVE™ technology enhances nutrient distribution and gas exchange, crucial factors for maintaining optimal cell growth and viability. Designed specifically for the scalable expansion of anchorage-independent cells for immunotherapy (Chimeric Antigen Receptor T-cells) but can be involved in MSCs production on microcarriers as well [28]. Silva et al. performed expansion of UC-MSCs on CultiSpher-S microcarrier inoculated to 2 L cell bag in small volume and kept static during the initial 24 h. After that UC-MSCs were expanded in rocking culture in 600 mL of culture medium. Overall, this system generates an expansion factor ranging from 12.3 to 24.8. Importantly, the dynamic conditions employed in the rocking culture did not negatively impact the quality of the expanded cells, as verified through various quality control measures, including viability, phenotype retention, and differentiation potential [29, 30]. Interestingly, another study found that MSCs aggregates spontaneously produced in a rocking bioreactor exhibit increased stemness, migratory properties, and angiogenic factor expression, thus offering increased therapeutic potential [31]. Overall, the Xuri™ Cell Expansion System W25 is a versatile and efficient tool for the expansion of MSCs, offering a reliable solution for both research and clinical applications.

## Scaling up of Cell Culture Vessels

Multilayered flasks are an essential part of large-scale MSCs production, offering a greater surface area compared to the traditionally used T-25, T-75, and T-175 flasks (Table 1), thereby reducing the extensive subcultures of MSCs. Generally, scale-up involves using larger bioreactors to increase production capacity, requiring extensive optimization to maintain cell quality in a larger volume. In contrast, scale-out expands capacity by adding more bioreactors of the same size, ensuring consistent conditions across units but needing more space and resources. The choice between scale-up and scale-out depends on factors like production goals, budget, and the ability to maintain cell consistency [32]. Nowadays, multilayered flasks are one of the most potent large-scale strategies, with a reported expansion ratio of 100-fold and even higher [33, 34]. On the other hand, as cell culture processes are scaled up to larger vessel sizes, maintaining a sterile and homogeneous culture environment throughout the production process becomes increasingly challenging [35].

**Table 1:**
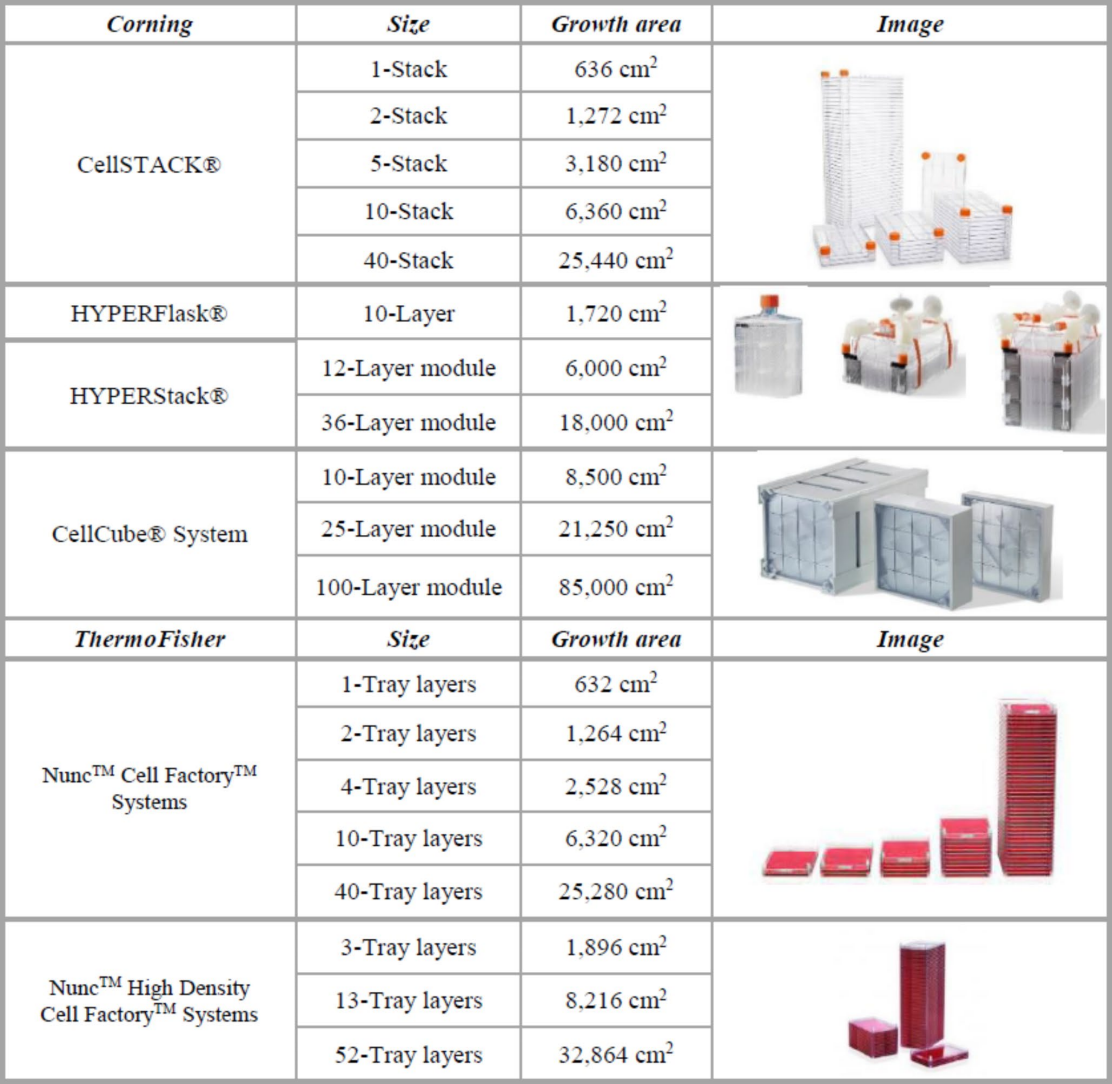
The growth area of different multilayer culture flasks from Corning and ThermoFisher

The CellSTACK^®^ multilayered flasks (Corning) and Nunc™ Cell Factory™ Systems (ThermoFisher) are examples of such vessels, consisting of multiple layers of cell culture surfaces stacked together in a single unit. Both of them are available in 5 sizes, ranging from 1 to 40 layers, and each layer provides a growth area of 636- and 632-cm^2^ [21]. For 2-chamber CellSTACK^®^ it was reported final cell yield of 2.84 × 10^8^ (WJ-MSCs (Wharton´s Jelly-MSCs) [33] and 4.69–5.65 × 10^7^ (BM-MSCs) [36] over the five days of culture. Comparing these two independent studies, there was a noted variance in the number of initially seeded cells ranging from 1000- to 4000- cells/cm^2^ resulting in a massive difference in calculated expansion ratio of 195.28- and 9.22-11.10- fold. Similarly, using 4-chamber Nunc™ Cell Factory™ two independent authors achieved different cell yields in harvest coresponding to 7.8 × 10^8^ and 2.5 × 10^8^ of expanded BM-MSCs [37, 38]. Finally, an 11-day culture of MSCs derived from dental pulp (DP-MSCs) in 5-chamber CellSTACK^®^ yielded 3.2 and 4.98 × 10^8^ cells cultured in FBS- and hPL-supplemented medium, respectively [34]. Overall, cell culture parameters like seeding density, culture media, certain additives, and length of culture affect the cell yield of a given system [39]. Moreover, Corning’s CellBIND^®^ surface treatment enhances cell attachment and growth by increasing the hydrophilicity of polystyrene cell culture vessels, making them more conducive for a wide range of cell types. This treatment ensures consistent, robust cell culture conditions, which is particularly beneficial for serum-free or low-serum media applications [32].

The HYPERFlask^®^ and HYPERStack^®^ vessels use a unique cells-medium-environment gas exchange technology via gas-permeable material that eliminates internal headspace within the vessel. Expanding MSCs derived from human umbilical cord matrix (UCM-MSCs) in 10-layer HYPERFlask^®^ (1720 cm^2^) seeded at a concentration 2 × 10^3^ cells/cm^2^ (3.44 × 10^6^ in total) in α-MEM supplemented with 15% human AB serum enabled the production of 4.5 × 10^7^ cells after 11 days in culture equal to the expansion ratio of 12.99 [40]. The HYPERStack^®^ consists of Stackettes– an individual cell culture chamber, comprised of the top plate and gas permeable film. Each Stackette possesses 500 cm^2^ of growth area, that are interconnected by two (liquid and air) manifolds to form a module with an open-air tracheal space between each stackette. As a result, the 12- and 36-layer modules (equal to 6000- and 18000-cm^2^) of HYPERStack^®^ have the same volumetric footprint as traditional 2- and 10-layer stacked culture vessels. Thus minimizing the culture space while fulfilling the metabolic needs of cultured cells [41]. Initial data performed by Sherman et al. revealed that a five-day expansion of BM-MSCs seeded at a density of 3 × 10^3^ cells/ cm^2^ into a 36-layer HYPERStack^®^ vessel can result in an average yield of 870 million cells in harvest (expansion ratio of 16.11) [42].

The Corning^®^ CellCube^®^ System offers a novel, robust, and scalable platform for adherent cell culture. Its modular design, comprising parallel 10, 25, or 100 polystyrene plates, provides a large growth surface area ranging from 8,500 to 85,000 cm^2^ in a compact footprint. Cells are seeded and cultured on both sides of each culture plate inside the CellCube^®^ System, which doubles culturing space. The seeding procedure requires optimization of attachment time and multiple vertical rotations of the system to inoculate cells uniformly. Moreover, a built-in peristaltic pump and single-use bioreactor ensure efficient continuous fluid recirculation for optimal gas and nutrient delivery. This perfusion-based design of the CellCube^®^ System creates an environment that closely resembles in vivo conditions thus increasing cell productivity. The provided information outlines the successful expansion of HEK293T and Vero cell cultures in the 100-layer CellCube^®^ module, demonstrating tight control of culture conditions and the use of metabolite analysis to determine harvesting time. Both HEK293T and Vero cell cultures expanded efficiently reaching yields of 1.4 and 1.1 × 10^10^ cells, respectively [43].

## Optimizing MSCs Manufacturing: Harnessing GMP-Compliant Serum and Serum-free Culture Media

Process validation is a crucial step in medicine development, during which the use of culture medium, additives, and materials that could compromise the safety of the product must be carefully evaluated. For instance, the use of animal-derived additives poses a risk of pathogen transmission, requiring thorough risk analysis and consideration of alternative additives. The selection of the appropriate culture medium is indispensable for MSCs expansion and quality, especially in large-scale production for clinical purposes. FBS has a longstanding history of utilization as a natural supplement rich in growth factors and nutrients, in numerous laboratory applications for the in vitro expansion of cells. However, employing FBS for the cultivation of MSCs intended for further use in therapeutic applications poses significant risks, primarily due to potential contamination with bovine viruses, prions, and zoonotic agents which can elicit immune responses in humans. In GMP-grade MSCs production, utilizing validated FBS intended for thorough inspection of the presence of immunomodulating agents, is crucial for upholding quality standards. Gamma irradiation treatment is considered one of the optimal approaches for preventing viral contamination in FBS, and it aligns with GMP processes [44–46]. Nevertheless, employing xenogenic (xeno)-free (XF) media, such as serum-containing media enriched with human-derived substances or serum-free and xeno-free media (SXFM), represents an optimal choice for GMP-compliant manufacturing of MSCs [47]. Incorporating human components into the culture medium instead of animal derivatives ensures compliance with GMP guidelines for cell expansion processes. Presently, humanized supplements, comprising human serum, umbilical cord blood serum, platelet-rich plasma, and hPL, are utilized as substitutes for FBS (Table 2.) [48]. Although autologous human serum is optimal for MSCs expansion, obtaining this in amounts sufficient to produce clinically relevant quantities of MSCs would be challenging [49]. However, pooled allogeneic human serum sourced from multiple donors could help overcome this limitation and facilitate large-scale production of MSCs [50]. Nonetheless, human-derived supplements (e.g. autologous human serum) have the next notable drawback, namely the significant variability observed from one batch to another. Although these variations could be mitigated by producing large donor pools per batch, they concurrently elevate the risk of infection caused by the possible presence of human viruses. Consequently, these hindrances can pose significant challenges to sufficient expansion of MSCs and related therapeutic efficacy in large-scale clinical trials [51]. To achieve a safe human product without using any animal products, researchers validated MSCs production under GMP conditions using hPL inactivated with psoralen or riboflavin as a substitute for FBS. This approach preserved the MSCs’ multipotent and immunomodulatory capacities [52, 53]. Mareschi et al. isolated MSCs from five bone marrow samples using hPL instead of FBS. They investigated a new hPL production method involving treating platelet pools with Ca-Gluconate to form a gel clot and mechanically squeezing it to release growth factors. The standard hPL (hPL-E), which is obtained through freezing/thawing cycles and heparin addition, was compared to the new hPL (hPL-S) had no platelets or fibrinogen but contained similar amounts of proteins and growth factors. hPL-S required fewer production steps to comply with GMP conditions, maintained stem cell markers, and showed significant differences, such as lower HLA-DR expression, making it an effective alternative for MSCs production [54]. At present, there are several commercially available virus-inactivated GMP hPL as well as final commercial products like Human Platelet Lysate (Life Science Production), Platelet Lysate MultiPLi (Macopharma), and PLUS™ GMP grade (Compass Biomedical Inc) [55]. Furthermore, contamination risks associated with hPL are effectively managed. For example, since 2018, all platelet concentrates in France have undergone psoralen treatment to mitigate the risks of microbial contamination [56]. Another method to eliminate potential pathogens involves gamma irradiation of hPL before its utilization [57]. However, a significant limitation associated with the manufacturing of hPL products is the necessity of ensuring an adequate supply of platelet donors [55]. A complementary option for the management of GMP-compliant media is the usage of synthetic, chemically defined media, such as BD Mosaic™ Mesenchymal Stem Cell Serum-Free (BD Biosciences), MesenCult™-XF (StemCell Technologies), MSC NutriStem^®^ XF (Biological Industries), Xerum free defined cell culture (TNC Bio BV), etc. The composition of the aforementioned media consists solely of known compounds, making them potential preferable alternatives to FBS. Nonetheless, they are particularly preferred for research purposes because they require continuous development and refinement to sustain crucial cellular functions such as adhesion, differentiation, immunomodulation, and other essential processes. The next group of media for optimized and reproducible MSCs production represents serum-free, xeno-free formulations with high-quality components, such as MSC-Brew GMP Medium (Miltenyi Biotec) and StemPro MSC SFM XenoFree™ (Invitrogen), etc [45, 51, 58, 59]. However, a key limitation of chemically defined media is the slow or limited MSCs proliferation and inadequate cell adherence to the substrate, due to a deficiency of attachment factors. Therefore, the usage of certain media requires pre-coating the plastic surface with specific substances to ensure optimal cell attachment and growth. On the other hand, media such as StemPro MSC SFM™ (Life Technologies), supplemented with human plasma derivatives, were not optimal for MSCs cultivation without coating [55, 58]. The MSC-Brew GMP Medium (Miltenyi Biotec) is the only SXFM on the market produced under GMP compliance, as it is bag-conditioned and intended for closed system culture, and requires no surface coating [55]. The most commonly utilized molecules, such as fibronectin, laminin, and peptides, are employed in surface modification to enhance adhesion by introducing positive surface charges to promote cell growth [17, 51].

Comparing the effects of diverse media types, supplemented with animal or human serum, versus serum-free media (SFM) yields notable results on MSCs characteristics. Dreher et al. demonstrated that AT-MSCs cultured in a medium supplemented with 10% human serum, compared to those cultured in 10% FBS, showed diminished presence in the lungs and livers of nonobese diabetic immunodeficiency mice after application. Conclusively, AT-MSCs cultured in the presence of human serum showcased intriguing variations in the downregulation of specific adhesion and extracellular matrix-associated molecules. They attributed that a reduction in the expression of integrin α6 (CD49f) may correlate with diminished adhesion to laminin [60, 61]. Nonetheless, the reduced entrapment of MSCs in the lungs could potentially mitigate the risk of pulmonary embolism [62]. The selection of cell expansion medium plays a pivotal role in influencing characteristics such as the proliferation kinetics of MSCs. Multiple experimental studies have unveiled notable effects of various media types on MSCs properties and culture dynamics. Wuchter et al. conducted a comparative analysis examining the effects of three GMP-compatible culture media for MSCs expansion: basal medium (Dulbecco’s Modified Eagle Medium (DMEM) - low glucose) supplemented with 10% pooled hPL, StemPro^®^ MSC SFM CTS (Invitrogen) (for human ex-vivo tissue and cell culture processing applications), and non-XF MSCGM™ (Lonza), supplemented with 10% FBS (research-only application). The most significant disparities emerged in the size and proliferation kinetics of BM-MSCs following extended cultivation (50 days). StemPro^®^ MSC SFM CTS notably enhanced growth, yielding MSCs with smaller, yet highly uniform morphology compared to others. BM-MSCs cultured in an hPL medium exhibited pronounced protrusions but were smaller compared to those cultured in FCS-containing MSCGM™. However, a slightly larger increase in growth was observed in MSCs cultured with hPL medium compared to culturing in MSCGM™ [63]. The promotion of MSCs expansion by platelet lysate also aligns with findings from other studies [64, 65]. Furthermore, Poloni et al. demonstrated that CD271-positive BM-MSCs, cultured in Iscove’s modified Dulbecco’s medium containing 10% pooled allogeneic human serum, exhibited comparable differentiation capacity and expression of surface and gene markers compared to those cultured in media supplemented with animal serum. Furthermore, the karyotype alterations were not observed. They also demonstrated a remarkable increase of cell production to clinically significant levels (10 × 10^8 MSCs) from 5 mL of BM aspirate in only 30 days, employing CD271-selected cells and pooled allogeneic human serum [66]. Moreover, the utilization of SFM has also had noteworthy impacts on the properties of MSCs. For instance, PRIME-XV SFM (Irvine Scientific) has been shown to enhance colony-forming abilities, boost osteogenic potential, and increase population doublings in human BM-MSCs. Therefore, it represents a superior alternative to animal serum [67]. Despite the similar osmolality of FBS-based medium and PRIME-XV SFM (0.31 and 0.29 Osmol/kg, respectively), SFM has a closer approximation to human physiological conditions and is therefore suggested to have a potentially better impact on cells [67, 68]. Furthermore, Aussel et al. demonstrated that SXFM, specifically MSC-Brew GMP Medium (Miltenyi Biotec), significantly impacts the clonogenic efficiency of MSCs. Specifically, colony size and density increased in the SXFM condition compared to traditional hPL medium for AT-MSCs and BM-MSCs [55].

It is noteworthy to mention that also other supplementary reagents used for harvesting and isolation of MSCs intended for clinical applications must fulfill all safety standards as well. Currently, most clinical studies use porcine-derived trypsin for cell detachment preparation. However, to optimize this procedure, usage of recombinant trypsins such as TrypLE (Gibco, Thermo Fisher Scientific) or TrypZean (Sigma-Aldrich), is advantageous due to their gentler effects on cells and absence of xenogenic proteins compared to trypsin of porcine origin. For enzymatic tissue digestion under GMP conditions, it is recommended to use GMP-grade collagenases instead of collagenase type I, as the latter may contain components that could potentially carry infectious agents [69, 70]. For these purposes, e.g. Collagenase NB6 GMP Grade (Nordmark Biochemicals) derived from Clostridium histolyticum is a high purity tissue dissociation enzyme intended for isolation stem cells. It encompasses proteins demonstrating enzymatic activities, encompassing collagenase class I and II, neutral protease, and clostripain. The optimal concentration of 0.4 PZ U/mL Collagenase NB6 and a digestion time of 3 h demonstrated a higher yield of MSCs derived from Wharton’s jelly at passage 0 [70]. Moreover, other types of collagenases are also used for tissue digestion: CLSAFA (Worthington) and Liberase MTF-S GMP Grade (Roche) [69]. On the other hand, another alternative replacement for trypsin and collagenase is Accutase (Life Technologies), a mixture of proteolytic and collagenolytic enzymes [71, 72]. However, mechanical procedures such as centrifugation, filtration, or micro-fragmentation offer alternative methods to circumvent safety concerns associated with collagenase usage [73]. According to the data gathered thus far, MSCs derived from bone marrow, adipose tissue, and umbilical cord emerge as the most commonly utilized cell sources in clinical trials [74]. Nevertheless, AT-MSCs offer an advantage for large-scale production due to their enhanced genetic and morphological stability during prolonged cultivation and faster proliferation compared to their BM-MSC counterparts from the same donors [75]. Furthermore, AT-MSCs demonstrate reduced differentiation potential after 25 passages, while the declining immunosuppressive effect of BM-MSCs was observed after just 7 passages [76, 77].

**Table 2:** Media utilized for GMP-grade MSCs production

*Media* * classification*		*Name of culture* *medium*	*Manufacturer*	*Limitations*	*Benefits*	*Ref.*
*Xenogenic serum media*		Basal medium (e.g. DMEM) supplemented with 10% irradiated FBS	Different vendors	Chemically undefined; Batch-to-batch variability; Inconsistent cell culture results	Source of growth factors, cytokines, and mitogens; Enhanced cell survival and proliferation	[44, 46]
*Non-xenogenic serum media or media containing human-derived substances*		Basal medium (e.g. DMEM) supplemented with 10% pooled allogeneic human serum	Different vendors	Batch-to-batch variability (potentially reduced by pooling); Risk for pathogen contamination; Less reproducible results and more presence of heterogeneous cell population	No risk of xenogeneic immune response	[51]
		Human Platelet Lysate	Life Science Production	Limited availability of platelet donations supplies; Batch-to-batch variability (potentially reduced by pooling); Less reproducible results and more presence of heterogenous cell population	No risk of xenogeneic immune response; Rich source growth factor; Enhanced proliferation capacity; Viral inactivated	[55, 59, 63]
		Platelet Lysate MultiPLi	Macopharma			
		PLUS™ GMP grade human platelet lysate	Compass Biomedical Inc			
*Serum- free and/or xeno- free media*	SF	TheraPEAK™ MSCGM™	Lonza	No xeno-free (contains bovine proteins including transferrin and albumin); Demand pre-coating the plastic surface	Chemically defined; Consistent quality across all batches	
	SF	BD Mosaic™ MSC SF	BD Biosciences	Most of them lack attachment factors; Demand precoating the plastic surfaces to support cell attachment; Most of them are used only for research purposes; Increases production complexity; Elevated production costs compared to FBS or hPL	Chemically defined; Consistent quality across all batches; Reduced risk of disease transmission; No risk of xenogenic contamination;	
	SF	StemPro™ MSC SFM	Invitrogen			
	SF	Xerum free defined cell culture	TNC Bio BV			
	SF, XF	MesenCult™-XF	StemCell Technologies			
	SF, XF	MSC NutriStem® XF	Biological Industries			
	SF, XF	PRIME-XV® MSC Expansion XSFM	Fujifilm Irvine Scientific			
	SF, XF	StemPro MSC SFM XenoFree™	Invitrogen		Consistent quality across all batches; Reduced risk of disease transmission	
	SF, XF	MSC-Brew GMP	Miltenyi Biotec	Elevated production costs compared to FBS or hPL	GMP-compliance; Packaged in bags; Compatible with closed system culture procedures; Not require coating	

*DMEM* Dulbecco's Modified Eagle Medium; *FBS* fetal bovine serum; *GMP* Good Manufacturing Practice; *hPL* human platelet lysate; *MSC* mesenchymal stem cell; *SF* serum-free; *SFM* serum-free medium; *XF* xeno-free; *XSFM* xeno-free and serum-free medium

## Cryopreservation of MSCs in Clinical Conditions

The application of cells or tissues in cell therapy has resulted in the creation of a new category of medicines for treating acquired or hereditary diseases. These are known ATMPs which are defined as therapeutic products where the primary biological action is performed by cells or tissues. Cryopreservation remains the only technique to preserve cells, including MSCs, for extended periods. It helps maintain cellular functional properties and allows for the aggregation of cells to achieve the necessary numbers for clinical use [78]. Compared to the cryopreservation of specialized cells like osteoblasts and chondrocytes, more factors must be considered for MSCs. Specifically, it’s crucial to evaluate the immunomodulatory and multilineage differentiation capabilities of MSCs after cryopreservation, cell viability, and phenotype. If these abilities are compromised, MSCs may fail to preserve the original immunomodulating and regenerative properties [79].

### Cryoprotecting Agents

**Fig. 2:**
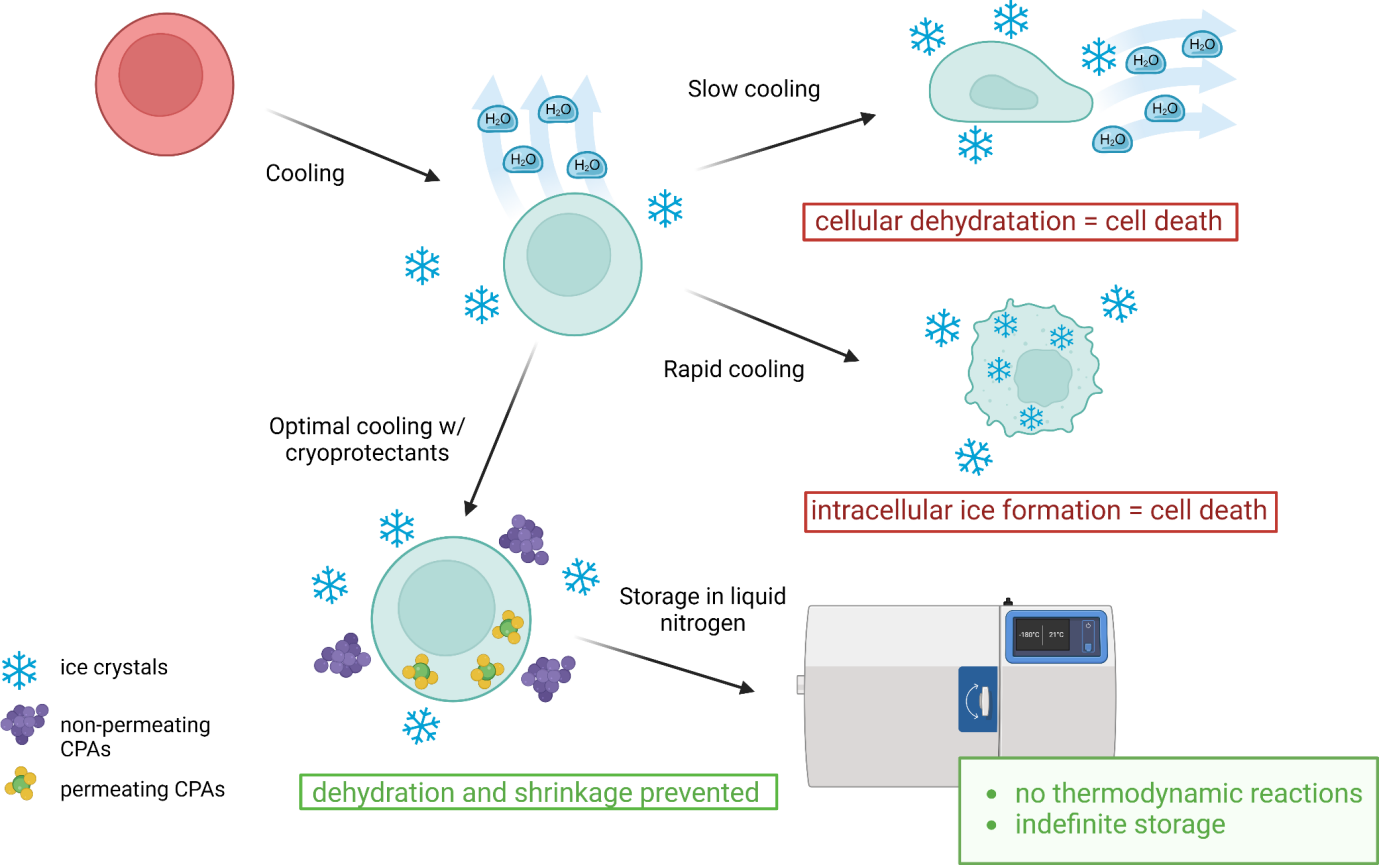
Cell cryopreservation mechanism as a crucial step for cell-based therapy manufacturing. Processes that lead to cell damage during cryopreservation involve specific mechanisms. To prevent this injury, it’s essential to add the right amount of appropriate cryoprotective agents (CPAs) to cell suspensions and apply optimal cooling rates. Created with BioRender.com

Cryoprotectors are agents capable of preventing biological objects from suffering freezing damage and ensuring their viability upon thawing. The protective action of cryoprotectors during freezing is based on their ability to create strong bonds with water molecules inside and outside the cell, which are stronger than the bonds between water molecules themselves. Additionally, they reduce salt concentrations, minimizing the risk of damaging the cells’ protein structures. Cryoprotectors also bond with the structural components of the membrane, protecting them from being damaged by ice crystals (Fig. 2) [80].

Various techniques have been developed for the cryopreservation of MSCs, such as slow freezing and vitrification. However, these methods have limitations. Firstly, even with cryoprotective agents (CPAs), MSCs preserved using these methods are still at risk of cryo-injury [81]. Secondly, CPAs like dimethyl sulfoxide (DMSO) may inadvertently induce the differentiation of MSCs into neuron-like cells. To address these issues, either optimizing CPAs use or developing new cryopreservation methods is necessary. Recent advances in micro/nanotechnologies offer promising improvements, potentially enhancing the efficiency of cryopreservation and overcoming the limitations of current methods. Innovations such as freezing cells encapsulated in nanoliter droplets on highly hydrophobic nano-rough surfaces and creating nontoxic nanoscale bio-inspired CPAs are particularly promising advancements in the field [82].

The established cryopreservation protocols for MSCs utilize DMSO. The residual trace amounts of DMSO, even after the washing step, pose a significant risk of adverse reactions in patients [83]. Additionally, DMSO has been linked to abnormal gene expression and the differentiation of MSCs. Therefore, developing a cryopreservation method for MSCs free of DMSO is highly desirable. Substances such as trehalose, plant proteins, and antifreeze protein mimics have been combined with reduced concentrations of DMSO to improve MSCs cryopreservation. These combinations have successfully inhibited ice recrystallization and protected cell membranes similarly to natural antifreeze proteins [84–89]. Several commercially available cryopreservative options are currently utilized in the cryopreservation of MSCs.

CryoStor^®^ CS2, CS5, and CS10 are commercially available GMP-grade cryopreservation media engineered for optimizing the preservation of cells and tissues in ultra-low temperature environments, ranging from − 80 to − 196 °C. These formulations provide a protective environment during freezing, storage, and thawing, enhancing cell viability and function while eliminating the need for serum and high-cytotoxicity agents. CryoStor specifically addresses molecular biological aspects of cells, reducing Cryopreservation-Induced Delayed-Onset Cell Death, thus improving post-thaw viability, particularly for sensitive cell types like MSCs and hepatocytes. The product line consists of CryoStor CS2 (2% DMSO), CS5 (5% DMSO), and CS10 (10% DMSO). The cryopreservation process involves critical steps. Initially, cells are prepared through mechanical or enzymatic dissociation, followed by centrifugation to create a cell pellet. The supernatant is removed while minimizing culture medium dilution. Cold CryoStor media is then added to achieve a concentration of 0.5–10 × 10^6 cells/ml, incubating at 2–8 °C for about 10 min. Nucleation is initiated by lowering the temperature to − 80 °C at a controlled rate of − 1 °C per minute, holding at this temperature for 15–20 min before initiating ice nucleation around − 5 °C. Alternatively, samples can be kept at − 20 °C for two hours, followed by − 80 °C. Once frozen, they can be stored below − 130 °C in liquid nitrogen or at − 80 °C for the short term. Thawing is critical and should occur rapidly in a 37 °C water bath. After thawing, the cell-CryoStor mixture requires dilution with culture medium at 20–37 °C. Post-thaw viability should be assessed 24 h later using assays such as Live/Dead fluorescent assays or metabolic assays like MTT, to carefully compare against non-frozen controls for accuracy [90].

Ho et al. proposed a study where canine AT-MSCs, modified to overexpress a therapeutic transgene, were cryopreserved with CryoStor10 for up to 11 months. Results indicated that cryopreservation did not compromise transgene expression, cell viability, or migration capacity. Notably, thawed MSCs exhibited comparable cytotoxicity to fresh counterparts, effectively treating canine patients with cancer and resulting in over 20 months of progression-free survival, highlighting the viability of cryopreserved MSCs in clinical applications [91]. Moreover, equine MSCs were cryopreserved in CryoStor^®^ CS10 and evaluated for their viability in ponies. Studies by Williams et al. involved administering these MSCs via i.v. after simulated chilled transport and directly after thawing, with monitoring for clinical and hematological changes. Results showed no adverse reactions or deviation in blood parameters. There was an increase in CD4 + and CD8 + lymphocyte populations post-injection, indicating a possible immune response. CS10 effectively maintained MSCs’ viability. Further research is warranted to explore immune interactions with different MSCs donors [92].

A recent paper compared two cryoprotectants: STEM-CELLBANKER™ (CB) and 10% DMSO, both in a xeno-free medium, for AT-MSCs and BM-MSCs. Both cryoprotectants maintained similar cell morphology and surface marker expression compared to non-cryopreserved MSCs. However, cryopreserved AT-MSCs had better viability with CB (90.4%) than with DMSO (79.9%). While population doubling time was comparable for AT-MSCs in CB and non-cryopreserved conditions, it was slightly increased with DMSO. BM-MSCs showed longer doubling times for both cryoprotectants. Importantly, both MSCs types preserved their differentiation capabilities after cryopreservation. In conclusion, CB better maintains the characteristics of AT-MSCs than DMSO, which could influence cell therapy applications [93]. According to the manufacturer, STEM-CELLBANKER^®^ DMSO Free GMP grade is an innovative cryopreservation solution designed for preserving various cell types without DMSO. Manufactured in compliance with GMP guidelines from JPN, EU, US, and PIC/S, its formulation is free from serum, animal-derived components, and DMSO, alleviating concerns about purity essential for research. STEM-CELLBANKER^®^ DMSO Free significantly enhances cell viability while preserving crucial features like cellular pluripotency and effective post-thaw proliferation. The solution offers remarkable batch-to-batch stability, ensuring consistent performance across lots. The manufacturer also claims users will appreciate its convenience, as it requires no preparation; cells can be frozen directly at -80 ℃, simplifying the cryopreservation workflow. It is also registered with the U.S. FDA Drug Master File, allowing customers to request a Letter of Authorization for regulatory submissions. Overall, this product represents a significant advancement in cryopreservation for those seeking DMSO-free solutions.

Human umbilical cord (UC) is a promising source of MSCs, despite being classified as solid tissue, UC offers advantages like multiple uses from a single donor. However, previous cryopreservation methods using animal or allogeneic serums resulted in lower cell proliferation in frozen-thawed UC-MSCs. Shimazu’s research team established an optimal cryopreservation technique using a serum- and xeno-free cryoprotectant, STEM-CELLBANKER. After a slow freezing process and subsequent thawing, the preserved UC yielded MSCs that maintained typical phenotypes, immunosuppressive abilities, and differentiation potential comparable to fresh cells, demonstrating clinical viability [94].

### Cryopreserving Methods and Available Systems

There are two primary methods for cryopreserving MSCs: slow freezing and rapid freezing/vitrification. Slow freezing, with a rate of 1 °C/min, is favored in clinics and research labs due to its low contamination risk and easier processing, allowing a large number of MSCs to be frozen in one vial with low concentrations of CPAs. Despite the risk of freeze injury, optimization of CPAs use is crucial to prevent ice crystal formation [82, 95]. Vitrification, involving the rapid transformation of cell suspensions from the aqueous phase to a glass state using liquid nitrogen, requires multimolar CPAs mixtures and stepwise introduction to minimize chemical toxicity. However, it risks osmotic damage and is less suitable for large volumes due to the need for manual handling and high skill [79, 96, 97].

A novel cryopreservation protocol was developed, using betaine and electroporation. Betaine, a natural osmoprotectant, is stable, non-toxic, and highly hydrophilic, which allows it to competitively bind water molecules and prevent ice formation [98, 99]. Electroporation, widely accepted for delivering non-permeable molecules into cells, creates temporary hydrophobic pores in the cell membrane under the electric stimulus, allowing molecule passage [100, 101]. This combined method exhibited universal applicability and maintained high post-thaw cell viability in various MSCs types, including human-derived UC-MSCs, mouse-derived BM-MSCs, and transgenic UC-MSCs [102].

For allogeneic “off-the-shelf” clinical applications, large quantities of frozen MSCs are needed. Cryopreservation at high cell concentrations minimizes the amount of DMSO infused, reducing infusion-related toxicities. Using CellSeal^®^ cryogenic vials, cells at 10 million/mL were frozen with 5% DMSO to -80 °C and stored in liquid nitrogen. AT-MSCs frozen using this system showed high functional viability upon thawing. This system enables easy bedside thawing and administration with minimal residual DMSO, eliminating the need for cell washing. Future studies will examine the engraftment potential of post-thaw AT-MSCs in vivo [103].

In Table 3, we list commercially available options for controlled rate freezing systems utilized in MSCs cryopreservation. These advanced freezing systems play a crucial role in ensuring the viability and functionality of MSCs during the cryopreservation process, allowing for controlled temperature profiles that prevent the formation of ice crystals and preserve cellular integrity. The table provides details on various models, including their specifications, features, and typical applications, helping researchers and practitioners make informed decisions when selecting the appropriate equipment for their MSC cryopreservation needs.

**Table 3:** An overview of commercially available controlled freezing systems used for cryopreservation of MSCs

Brand/ Model	Manufacturer	Key Features	Temperature range	Capacity	Application	Additional Notes	Ref.
CoolCell	Corning	Passive cooling, without alcohol or any fluids	-80 °C	6 to 30 vials of 1–10 mL volume	Ideal for small-scale cryopreservation	Easy to use, no electricity required	[104, 105]
Mr.Frosty	Thermo Fisher Scientific	Passive cryopreservation, consistent cooling rates	-80 °C	12 to 18 tubes	Suitable for small sample sizes	Simple design, economical solution, requires only 100% isopropyl alcohol and a mechanical freezer	[105]
Kryo570	Planer	Controlled-rate freezing with programmable options	up to -180 °C	784 x 1.0 mL − 2.0 mL or 244 x 5.0 mL vials	Large-scale cryopreservation	Software for data logging, customizable protocols	[106]
CryoVita	Antech Scientific	Liquid nitrogen vapor-phase freezer	up to -180 °C	380–1185 x 2mL vials	High-throughput applications	Temperatures are monitored by Type T thermocouples, eliminating lag time and providing “real-time” responsiveness	[107]
CryoMed	ThermoFisher	Modular design for flexibility in applications	up to -180 °C	up to 1200 vials	Ideal for research and clinical labs	Customizable for multiple user needs	[108]
Via Freeze	Cytiva	Supports cryopreservation with precise temperature control, Data logging feature for temperature monitoring	-80 °C or lower, depending on specific models and configurations	Varies by model; typically accommodates multiple samples or large volumes for laboratory use	Used in cell therapy, regenerative medicine, and biobanking	Integrated with other lab equipment, relevant accessories, and consumables available	[109]

Regarding the cryopreservation of MSCs suspensions, it is essential to focus on standardizing the evaluation of cell culture quality. Particularly, the consistency of the assessment criteria both before and following cryopreservation is crucial. By comparing similar metrics before and after freezing, we can accurately determine the effect of cryopreservation on the cell culture. Additionally, it is important to recognize that the cell culture must undergo a restoration or acclimatization process post-cryopreservation. Several studies have shown that without this acclimatization phase, fully recovering the therapeutic potential of the cryopreserved stem cells is not achievable [110, 111].

It is important to recognize that various complex cell-based products can differ greatly, meaning that a single cryopreservation protocol may not be suitable for all. Consequently, creating a separate protocol for each product complicates standardization efforts. The cryopreservation of complex biomedical cell-based products thus poses a challenging and contentious issue. However, research suggests that the concept is achievable and warrants more in-depth investigation. Another aspect that hinders the standardization of cryopreservation is the significant differences in technical equipment across laboratories. Typically, each lab establishes its cryopreservation protocol based on its specific technical capabilities and available reagents. Therefore, an integrated approach is necessary to standardize the cryopreservation process. Nonetheless, it should be feasible to compile general guidelines to enhance the cryopreservation process, minimizing stress on the cells. The authors hope that this review aids in organizing and selecting methods for the cryopreservation of MSCs, MSCs-containing tissues, and related biomedical cell-based products.

## Conclusion

One limitation of current large-scale expansion platforms for manufacturing clinical-grade MSCs is the variability in cell yield and quality between different systems and methodologies. While various bioreactors and culture vessels have been developed to address the need for scalable production, there remains a lack of standardization in terms of culture conditions, cell handling techniques, and quality control measures. This variability can result in inconsistencies in the final product, affecting its therapeutic efficacy and safety. Additionally, the complexity and cost associated with some of these platforms may limit their widespread adoption, particularly in resource-constrained settings or for smaller-scale production needs.

Moving forward, efforts should focus on standardizing protocols and optimizing large-scale expansion platforms to ensure reproducibility, consistency, and cost-effectiveness in the manufacturing of clinical-grade MSCs. This could involve the development of more user-friendly and modular systems that allow for seamless integration into existing manufacturing processes, as well as the implementation of advanced monitoring and control technologies to ensure precise regulation of culture conditions. Furthermore, there is a need for continued research into novel culture substrates, media formulations, and growth factors that can enhance cell proliferation, maintain stemness, and promote therapeutic potency. Cryopreservation of MSCs is also critical for maintaining their viability and therapeutic potential in clinical applications. While DMSO is commonly used as a cryoprotective agent, it presents risks such as toxicity and unwanted differentiation, prompting the development of alternative cryopreservation methods and agents and commercially available GMP-grade cryopreservation media. Also advances in controlled-rate freezing systems have improved the post-thaw viability of MSCs. Collaborative initiatives involving industry, academia, and regulatory bodies will be crucial for accelerating the translation of MSCs-based therapies from the laboratory to the clinic. Ultimately, addressing these challenges and advancing large-scale manufacturing capabilities will be essential for realizing the full potential of MSCs-based regenerative medicine and meeting the growing demand for cell-based therapies.

## Data Availability

Not applicable.
